# Hippocampal formation volume, its subregions, and its specific contributions to visuospatial memory tasks

**DOI:** 10.1590/1414-431X20209481

**Published:** 2020-07-24

**Authors:** T. Shavitt, I.N.S. Johnson, M.C. Batistuzzo

**Affiliations:** 1 Departamento de Psiquiatria, Instituto de Psiquiatria, Hospital das Clinicas, Faculdade de Medicina, Universidade de São Paulo, São Paulo, SP, Brasil; 2 Child Study Center, Yale School of Medicine, New Haven, CT, USA; 3 Curso de Psicologia, Faculdade de Ciências Humanas e da Saúde, Pontifícia Universidade Católica de São Paulo, São Paulo, SP, Brasil

**Keywords:** Hippocampal formation, Magnetic resonance imaging, Structural neuroimaging, Visuospatial memory, IQ

## Abstract

Visuospatial memory (VSM) is the ability to represent and manipulate visual and spatial information. This cognitive function depends on the functioning of the hippocampal formation (HF), located in the medial portion of the temporal cortex. The present study aimed to investigate whether there is an association between the volume of the HF and performance in VSM tests. High-resolution structural images (T1) and neuropsychological tests evaluating VSM were performed on 31 healthy individuals. A VSM index was created by grouping 5 variables from 5 tasks (4 from the CANTAB battery and 1 from the Rey-Osterrieth Complex Figure test). Multiple linear regression models using the volumes of HF subregions as independent variables and the VSM index as the dependent variable were conducted to test the hypothesis that memory performance could be predicted by HF volumes. We also conducted analyses to explore the role of covariates that may mediate this relationship, specifically age and intelligence quotient (IQ). We found significant associations between the hippocampal subregions of the left hemisphere and the VSM index (F(7,22)=2.758, P=0.032, *R* ^2^_a_=0.298). When IQ was accounted for as a covariate, we also found significant results for the right hemisphere (F(8,21)=2.804, P=0.028, *R* ^2^_a_=0.517). We concluded that the bilateral hippocampal formations contributed to performance on VSM tasks. Also, VSM processing is essential for a diverse set of daily activities and may be influenced by demographic variables in healthy subjects.

## Introduction

Memory is a cognitive function defined as the ability to recognize, retain, fix, and evoke past experiences (1). It is essential to humans since the ability to learn from experiences has probably developed as an adaptive function throughout human evolution (2). Also, memory can be divided and classified in different ways according to specific characteristics or its neurobiology. Visuospatial memory (VSM) is one aspect of memory that consists of temporal representations of visual and spatial information (3). VSM is historically related to the functioning of the right cerebral hemisphere (4). Since visuospatial abilities are not necessarily influenced by language, they generally involve the retention and/or manipulation of visual images - mental representations of objects and the spatial relationships between objects. Visuospatial abilities range from the momentary visual perception of an object to the ability to imagine a change in the object or the addition of other objects. These abilities also range from spatial orientation perception to route planning (5). VSM is crucial for our interaction with the world, since it is related to the most diverse daily activities, from tasks such as the simple visual perception of objects in space to the capacity for orientation and navigation (6).

Together with other brain regions, the hippocampus forms the so-called hippocampal formation (HF), a brain structure located in the medial portion of the temporal cortex that participates in the brain circuits from which memory is derived (7). The HF is a C-shaped structure that, in humans, is approximately 5 cm in length (7). Based on its extrinsic connections, this brain area receives a large amount of sensory information that is mainly channeled through the entorhinal cortex (8). The entorhinal cortex is another part of the HF. The HF is also made up of the dentate gyrus, the subicular plexus, and the hippocampus itself, which is composed of four main parts: CA1, CA2, CA3, and CA4 (9). These parts are given this nomenclature because they are part of the cornu ammonis (CA) or Ammon's Horn. In the literature, it is common to divide the hippocampus into anterior and posterior subregions that represent different functions. The posterior portion seems to be involved in the use of previously learned spatial information, while the anterior region appears to be more involved in coding new environmental layouts (10). Furthermore, the dorsal (posterior) hippocampus appears to be more related to the topic of interest in the present study, visuospatial memory, since its neuronal cells trigger during spatial orientation tasks (11). The right hemisphere is believed to be more related to visuospatial memory compared with the left hemisphere which, in turn, is more strongly associated with the memory of verbal information (3).

A series of studies by Maguire et al. investigated the relationship between visuospatial memory and hippocampal volume using magnetic resonance imaging (10,12,13). The results of the first study indicated higher hippocampal volume (bilaterally) in taxi drivers compared to healthy controls since taxi drivers experience extensive memory training (10). The results obtained in the second study confirmed the data from the first study and emphasized the importance of segmentation of the hippocampus into its subdivisions for more precise and specific outcomes (13). The third study in the series compared the hippocampal volume of taxi drivers with bus drivers, who had the same amount of experience in the profession and the same level of stress, but differed in the routes they drove. This study confirmed once again the results obtained previously and demonstrated that spatial knowledge and spatial navigation capacity - not the level of stress or other possible factors - are associated with the gray matter volume of the hippocampus (12). In addition, a more recent study was conducted in 2012 in Norway to test the hypothesis that greater hippocampal volume would be associated with higher scores on memory tests, and that this would be related to hippocampal subdivisions and their specific functions. The results indicated that a larger left hippocampal volume is associated with improved verbal memory performance. More specifically, a higher volume of the CA2/3 and CA4/dentate gyrus regions was associated with greater ease in recalling requested words (14).

In general, both studies with London taxi drivers and the Norwegian study contributed to the investigation of the relationship between the volume of the hippocampal formation and performance on memory skills. The studies with taxi drivers focused on the relationship between visuospatial memory and the volume of the posterior hippocampus and the right hippocampus. The Norwegian study, on the other hand, presented valuable information about the left hippocampus and the relationship between its volume and verbal memory. Both studies showed that a general relationship between the volume of the hippocampal formation and performance in memory tasks is very likely.

The general objective of this study was to evaluate whether there is a relationship between the volume of the bilateral hippocampal formation (especially the right hemisphere) and its subdivisions with visuospatial memory task performance. We attempted to account for covariates that may interfere with this relationship, such as age and intelligence quotient (IQ). Our study aimed not only to replicate the previous findings, but also to introduce methodological innovations, such as analyzing the association between the volume of hippocampal subregions and visuospatial memory (mainly in the right hemisphere) using memory tests not used in previous studies, especially computerized tasks. We hypothesized that an increase in the volume of certain subregions of the hippocampal formation is correlated with better performance on memory tasks. Secondly, we also expected that certain demographic variables such as age, IQ, or sex would be associated with performance on memory tests. Therefore, our statistical analysis attempted to control for these covariates.

## Material and Methods

We conducted a cross-sectional study: thirty-one subjects were assessed by clinical and demographic evaluation, neuropsychological evaluation, and structural magnetic resonance imaging (MRI) scans. Participants were recruited at the Institute of Psychiatry of the Clinical Hospital at the Medical School of the University of Sao Paulo, as most of the participants worked at the hospital. They attended three evaluation sessions: two at the Institute of Psychiatry for testing, one neuropsychological evaluation lasting approximately 2 h and 30 min and a demographic and clinical evaluation that lasted about 2 h, and one at the Institute of Radiology, lasting approximately 1 h, for neuroimaging.

The clinical and demographic evaluation used interviews and scales that evaluate for the presence of psychiatric disorders, previous psychiatric treatments, and symptoms of depression and anxiety in a dimensional way. For the characterization of psychiatric disorders, we used the Structured Clinical Interview for DSM-IV Diagnosis (SCID) (15,16).

The neuropsychological evaluation included evaluations of IQ and visuospatial memory (VSM). The Wechsler Abbreviated Scale of Intelligence (WASI) (17,18) was used to assess IQ. This scale is composed of four subtests: Vocabulary, Block Design, Similarities, and Matrix Reasoning, which evaluate various cognitive aspects, such as verbal knowledge, visual information processing, spatial and nonverbal reasoning, and fluid and crystallized intelligence. Visuospatial memory was evaluated with five tests, described in later sections.

Finally, MRI was used in this study to integrate cognitive tests with their biological bases, since it provides information about the structure of cerebral areas (such as volume) involved in specific cognitive functions. Anatomical regions of interest (ROIs) were defined based on a previous similar study (19), in addition to the specific hippocampal subregions for which FreeSurfer (v6.0.0) provided automatic segmentation in its output. A Philips Achieva 3 Tesla scan (The Netherlands) was used, with a 32 channel head-coil. The pulse sequence used to obtain the structural image in high definition had a duration of 05'58''7'''. The parameters used for this 3D acquisition are: T1, voxel dimensions of 1 mm^3^ (isotropic), repetition time (TR) 7.0 ms, echo time (TE) 3.2 ms, 240×240 matrix, system (sensing encoding - SENSE) of 1.5, field of view (FOV) of 240 mm^2^, and flip angle of 8°.

All T1 images were visually inspected as part of quality control procedures, looking for artifacts that could interfere with FreeSurfer segmentation. This verification was performed with Mango software (Lancaster, Martinez; http://www.ric.uthscsa.edu/mango).

### Participants

This study evaluated 31 healthy subjects, of whom 11 were male and 20 were female, with ages ranging from 20 to 63 years. The Intelligence Quotient (IQ) had to be >70 for a subject to be included in our study (i.e., >2.2th percentile, thus excluding intellectual disability from the sample). We did not include potential participants who demonstrated, from the start, an inability to adhere to the study (lack of motivation, interest, or availability) or participants who had any psychiatric disorder, psychotic symptoms, epilepsy, history of head trauma, prior neurosurgery, or any other antecedents of neurological disease.

We excluded participants with a history of significant substance use, suicidal ideation with lethal intent, suspected suicide attempt, current pregnancy, contraindication to MRI (pacemaker, metal implants, intracranial metal clip, cochlear implant, orthodontic appliance, etc.). We also excluded participants whose structural imaging demonstrated lesions or clinically significant structural changes in the cerebral parenchyma.

### Materials

Besides the demographic and clinical evaluations, using the interviews and scales described above, the neuropsychological evaluation included the evaluation of IQ, using methods described above, and VSM. VSM was evaluated with five tests: the first one is a classical measure used in neuropsychological practice, the Rey-Osterrieth Complex Figure test, and the others are four computerized tests belonging to the Cambridge Neuropsychological Automated Battery Test (CANTAB) battery. The CANTAB tests have been used highly and their validity and reliability have been carefully assessed (20,21). The tests are described below. For further information, please consult the supplementary materials (Supplementary Figure S1).

#### Rey-Osterrieth Complex Figure (ROCF) test (22,23)

This task consists of a complex geometric figure composed of a large rectangle, horizontal and vertical bisectors, two diagonals, and geometric details internal and external to the large rectangle. The figure was displayed horizontally, and the participant was asked to copy it onto a blank sheet. After that, the participant was asked to draw the figure using only his/her memory, after 3 min (immediate recall) and then after 30 min (delayed recall). Scores on ROCF vary between 0 and 36 - each element of the figure can assume a value between 0 and 2 and there are 18 items (24).

#### Spatial Span (SSP)

Analogous to the Corsi block-tapping subtest from the Wechsler Memory Scale - Revised (WMS-R) (25), this task lasts approximately five minutes and consists of white squares appearing on the computer screen, some of them changing briefly in color, following a specific sequence. The participant was asked to click on the squares that changed color, in the same order as the sequence. The number of squares in the sequence increased progressively, from two at the start of the test to nine at the end of it. The sequence varied throughout the test, and the participant had up to three chances to complete the sequence at each difficulty level. When the participant missed an entire level, the test ended. The variables used were the longest sequence completed (span length) and the total number of errors.

#### Spatial Span Indirect (SSP-I)

The same procedure as SSP, but the subject had to respond in the reverse order that the sequence was initially presented in. The same variables (span length and the total number of errors) were used.

#### Delayed Matching to Sample (DMS)

The completion time of this task was seven minutes, and it relied on a complex model figure shown to the participant, followed by four similar figures, which appeared at the same time as the model figure, or after a short period of time (0, 4, or 12 s), alternatively. The participant must click on the figure that exactly matches the model. Outcome measures included the number of correct patterns selected.

#### Spatial Recognition Memory (SRM)

This is a test of visual-spatial recognition memory in a two-alternated forced choice paradigm. It lasted five min and the participant was presented with a white square, which appeared in a sequence at five different locations on the screen. In the recognition phase, the participant saw a series of five pairs of squares, one of which was seen at a location previously seen in the presentation phase. The other square was in a location not seen in the presentation phase (distractor stimulus). This procedure was repeated three times and the sum of the total number of hits was used as one of the dependent variables.

### Data analysis

Processing and analysis of the T1 structural images were performed using the latest version of FreeSurfer (v 6.0.0) (26,27), which allows for, among other functions, the automatic segmentation of subcortical regions, especially the region of interest in this study (hippocampal formation). Briefly, FreeSurfer can separate the brain structures from a previously divided brain template, attempting to apply these pre-divisions to the T1 images of the study participants. Although the software automatically provides volumes of the hippocampal formation, a manual check was necessary to ensure quality control of this segmentation. This meant that each image was manually inspected to ensure the quality of the data provided by the program. Images that were of poor quality or had errors in segmentation required new MRI evaluation. No editing was required. Moreover, in this study, we used FreeSurfer's functionality, which segments the hippocampus into subfields (28).

The seven hippocampal subregions selected for analysis were: CA1, CA3, CA4, the granular cells of the molecular layer of the dentate gyrus, pre-subiculum, subiculum, and parasubiculum. Certain regions were not analyzed, such as the fimbria (region of white matter), hippocampal fissure, or the transition area between the hippocampus and amygdala, and the molecular layer. These same regions were excluded from the analyses in a previous study. Furthermore, we also used the hippocampal subdivision between tail (posterior) and head (anterior) encompassing specific subregions, which is provided by the most recent version of the FreeSurfer software (v 6.0.0) (28).

Descriptive statistical analysis of the study's demographic data was determined using the mean, standard deviation, and frequency of the data obtained. All variables were tested for normal distribution using the Kolmogorov-Smirnov test. Once normality was established in behavioral data (memory) and hippocampal volumes, these variables were then analyzed using Pearson’s correlation and corrected for multiple comparisons using the Bonferroni method. After this analysis, two multiple linear regression analyses (forced entry method) were performed in which the dependent variable was the value obtained from the performance variable in the visuospatial memory tests (visuospatial memory index, see below) and the independent variables were: 1) the seven volumetric measurements of the different subregions of the hippocampal formation for each hemisphere; and 2) the volumetric measurements of the head and tail of the hippocampus for each hemisphere.

Before performing the inferential analysis reported above, we conducted an exploratory factorial analysis (EFA) in order to represent VSM as one dependent variable. Therefore, EFA was performed with raw scores from the seven variables evaluated by the neuropsychological tests: two variables were selected from both the SSP and SSP-I tests and one variable was selected from each of the Rey Figure, DMS, and SRM tests. This procedure allowed us to investigate which of the seven variables were related to VSM, and which were not directly related to this factor. After completing the EFA, we separated and normalized (Z-score) by each of the five variables that composed this VSM. The average of these five Z-scores was used to calculate the VSM index. This meant that different VSM indices were made for each subject, so that this value could be correlated with the volume of the hippocampal subregions for each subject. The statistical significance level adopted for this study (alpha) was 0.05, and all statistical analyses were performed using JASP (JASP Team, Version 0.8.6, 2018, <https://jasp-stats.org/2018/02/28/now-jasp-0-8-6/>).

### Ethical considerations

This study was carried out with data extracted from a project conducted at the Institute of Psychiatry at the Hospital das Clínicas of the Medical School of the University of São Paulo (IPq-HC-FMUSP, Brazil) and approved by the Ethics Committee for Analysis of Research Projects HCFMUSP (CAPPesq - opinion number: 1,015,347, dated 08/04/15, online registration 12047) and the committees of the Departments of Psychiatry and Radiology. All participants signed the free and informed consent form, making it clear that their participation in the study was voluntary and that they consented to the use of their imaging data and their performance on neuropsychological testing for this study.

## Results

### Demographic and neuropsychological data

Table 1 shows the initial data obtained from the demographic and neuropsychological evaluations for the subjects participating in this study. Regarding the demographic data, the average age of the subjects was 35 years, ranging from 20 to 63 years. All subjects had from 12 to 20 years of schooling and average IQ was at the upper-middle range (mean 111.4). Most often, the MRI occurred after neuropsychological evaluation. The length of time between neuropsychological assessment and MRI examination was on average 170 days.

**Table 1:** Demographic and neuropsychological data from 31 healthy controls who had data from magnetic resonance imaging and neuropsychological testing.

Variable	Average/Frequency	Standard deviation/ Percentage	Range
Age	34.9	11.5	20–63
Gender (male)	11	35%	–
Laterality (right-handed)	29	94%	–
Years of schooling	16.1	2.6	12–20
Total IQ	111.4	12.8	78–134
SSP Span length	6.87	1.25	4–9
SSP Total errors	15.7	6.26	7–35
SSP-I Span length	5.77	1.20	4–9
SSP-I Total errors	10.8	4.79	4–17
SRM Sum of correct clicks on valid blocks	11.0	2.03	6–14
DMS Number of correct patterns selected	13.1	1.52	11–15
Rey Figure Delayed recall	22.5	5.57	5–36

SSP: Spatial Span test; SSP-I: Special Span test (Spatial Span) in inverse mode; SRM: Spatial Recognition Memory test; DMS: Delayed Matching to Sample.

**Figure 1:**
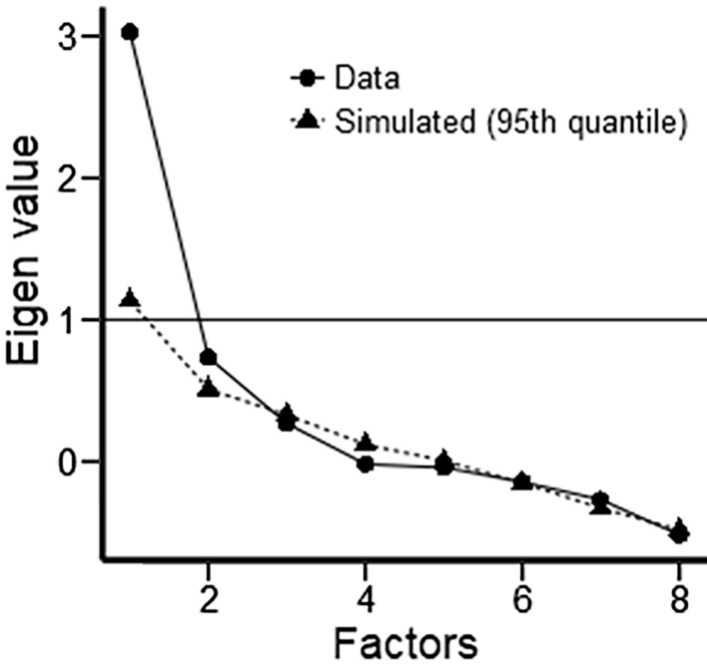
Exploratory factor analysis indicating that just one factor is the best solution.

An EFA was performed to investigate how many factors would effectively explain the set of neuropsychological variables (seven) evaluated in this study. The graph from the EFA, which indicates that one variable that represented the VSM data was the best solution, is shown in Figure 1. Table 2 shows the factorial load of each of the five variables that composed the VSM index (all factor loadings higher than 0.650).

**Table 2:** Factor loadings for each variable that composes the visuospatial memory index.

	Factor 1	Uniqueness
DMS Total correct (all delays)	0.868	0.246
Rey Figure score in the total late recall	0.700	0.510
SRM Sum of correct clicks on valid blocks	0.708	0.498
SSP I Span length	0.656	0.569
SSP Span length	0.855	0.270
SSP Total errors	–	0.958
SSP I Total errors	–	0.975

Analysis performed with exploratory factor analysis. SSP: Spatial Span test; SSP-I: Special Span test (Spatial Span) in inverse mode; SRM: Spatial Recognition Memory test; DMS: Delayed Matching to Sample.

### Hippocampal volume data

**Figure 2:**
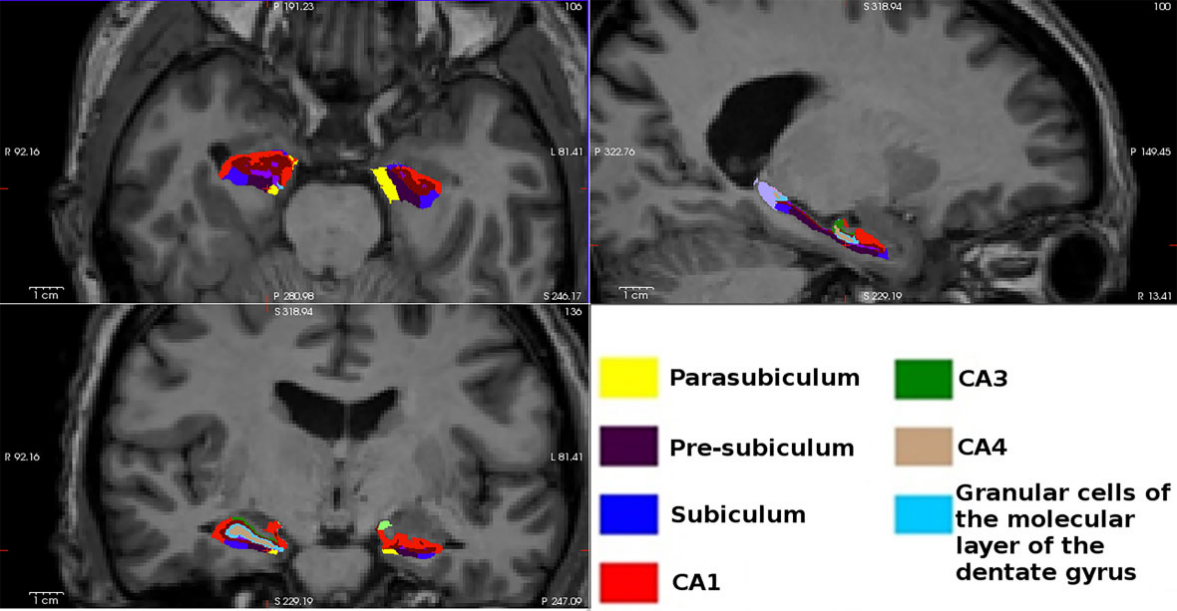
Segmentation of subregions of the bilateral hippocampal formation by FreeSurfer (v 6.0.0) software in sagittal (upper right), axial (upper left), and coronal (lower) magnetic resonance imaging views of the brain of a study participant.

After segmentation, all variables that represented volume data presented a normal distribution (Figure 2 contains a representation of the hippocampal formation and its division into subregions). The average size of the right hippocampal formation was 3847 mm^3^ and of the left, 3410 mm^3^.

### Relationship between hippocampal formation volume data and neuropsychological data

The total volumes of the HF did not correlate with the VSM index. Similarly, we did not find a correlation between the hippocampal tail and head volumes and the VSM index (Table 3). As a second step, the volumes of the tail and head of the hippocampal formation were selected as independent variables (separately for left and right hemispheres), which were used in multiple linear regression models to predict the VSM index. Contrary to our initial hypothesis, we did not find any positive correlation between these hippocampal regions and the VSM index for both the right (P=0.149) and left (P=0.598) hemispheres.

**Table 3:** Correlation coefficients of the visuospatial memory index with hippocampal subregion volumes.

	Left Hemisphere	Right Hemisphere
Hippocampal formation tail	–0.097	–0.115
Hippocampal formation head	0.193	0.067
Total hippocampal formation	0.095	0.014
CA1	0.181	–0.093
CA3	–0.089	–0.058
CA4	–0.055	–0.067
Granular cells of molecular layer of dentate gyrus	0.028	–0.057
Presubiculum	0.222	0.317
Subiculum	0.131	0.172
Parasubiculum	0.316	0.420^a^

Although the parasubiculum correlated with the visuospatial memory index (r=0.420, P=0.021), when multiple comparisons were controlled for (Bonferroni method, i.e., P<0.007), it was no longer significant.

Finally, to examine more specific subdivisions, seven subregions of the hippocampus (bilaterally) were selected. Table 3 describes these subregions, along with the correlation coefficient values of the VSM index, for each hemisphere. Although the parasubiculum correlated with the VSM index (r=0.420, P=0.021), when multiple comparisons were controlled for (Bonferroni method, i.e., P<0.007), the correlation was no longer significant. Thus, to test the effect that all subregions collectively had on the VSM index of healthy subjects, a multiple linear regression model was utilized to analyze the hippocampal subregions. For the left hemisphere, the model presented statistical significance (F (7,22)=2.758, P=0.032, R^2^=0.467, *R* ^2^_a_=0.298), and the granular cells of the molecular layer of the dentate gyrus (β=2.694, P=0.023) and CA4 (β=–3.194, P=0.010) were subregions whose volumes were predictive of the VSM index (the other predictors can be visualized in Supplementary Table S1). For the right hemisphere, the model did not present statistical significance (F (7,22)=1.835, P=0.131, *R* ^2^_a_= 0.168), and no subregion contributed to predicting the VSM index (Supplementary Table S1).

### Effect of covariates (age and IQ)

When age was correlated with the head and tail regions and the seven bilateral subregions of the hippocampus, there were no positive correlations. The right parasubiculum, however, was the only region negatively correlated with age (r=–0.510, P=0.003, corrected for multiple comparisons). We did not find a significant correlation between age and VSM index. When correlating IQ with these same variables, we found a positive correlation with the VSM index (r=0.592, P<0.001), but not with the volumes.

When the subjects' age and IQ were entered as covariates (separately) in multiple linear regression models for the right hemisphere using the VSM index as the dependent variable, the model demonstrated no significance for age (F (8,21)=1.539, P=0.203, *R* ^2^_a_=0.130), but did demonstrate significance for IQ (F (8,21)=2.804, P=0.028, *R* ^2^_a_=0.517). In the latter comparison, IQ was the only predictor associated with the dependent variable (P=0.019, β=2.534) and there was no substantial change in the significance level for the other subregions.

For the left hemisphere, in turn, when age was inserted into the model, this multiple linear regression model using the VSM index as the dependent variable remained significant (F (8.21)=2.680, P=0.034, *R* ^2^_a_=0.317) and both the CA4 subregion and the granular cells of the molecular layer continued to be predictors of the dependent variable (Ps = 0.023 and 0.050; β=–2.829 and 2.330). When IQ was inserted into the model as a covariate (instead of age), it continued to show statistical significance (F (8.21) *R* ^2^_a_=3.160, P=0.016, =0.373), although IQ did not account for the representation of VSM as one dependent variable (P=0.071, β=0.408).

Please consult the supplementary materials for tables that better illustrate the findings that were significant in the overall model and that further clarify which components were included in the multiple linear regression model (Supplementary Tables S1 and S2).

## Discussion

In this study, an exploratory factor analysis revealed one single variable composed of different features of VSM variables: short-term visual recognition memory (DMS and SSP), working memory (SSP-I), recognition memory (SRM), and episodic memory (Rey Figure). Thus, the VSM index that resulted from the EFA covered the common aspects of the visual spatial memory that was assessed by these tests. We did not find a correlation between the VSM index (Z-score) and the total volumes of the bilateral hippocampal formations. However, a more detailed model, integrating information from all seven subregions was able to predict the VSM index from the volumes of the subregions of the left hemisphere. This result did not support our initial hypothesis that the association would be stronger in the right hemisphere. This hypothesis followed from the results of the British studies (12), showing that taxi drivers (subjects with training in visuospatial skills) have larger right hippocampal volumes, compared with controls (particularly in the posterior region).

However, although we expected the association with VSM to be stronger in the right hemisphere, the initial hypothesis also predicted that left hemisphere volumes would be associated with performance on memory tasks, which was indeed demonstrated by our results. This is thought to occur because hippocampal formations are bilaterally involved in the processing of visuospatial memory in general (10). Following from this, it is possible to think about the participation of both hemispheres as important in VSM processes, although more specific functions may be different depending on laterality. Although it was possible to predict the VSM index only with left hemisphere volume data, this does not mean that the right hemisphere does not participate in this cognitive function. After all, there are numerous findings relating the right hippocampus to visuospatial memory (10,12,13,29).

Moreover, another difference between our study and the British study series is that our study found no significant correlation between hippocampal regions such as the tail and head and the VSM index. Studies with British taxi drivers predicted a positive association between the volume of the posterior portion of the hippocampus and better memory skills (10), which was not found in our study due to methodological differences and limitations of this study, which are discussed further in the following paragraphs. Nevertheless, when more specific subregions of the left hippocampal formation were analyzed within the regression model, the CA4 subregions and granular cells of the molecular layer of the dentate gyrus were shown to be predictors of the visuospatial memory index. It makes sense that these two regions showed significance since the CA4 subregion is considered by many authors to be a part of the dentate gyrus. They are located near each other, have similar cytoarchitecture, and are involved in related functions (7). The dentate gyrus is the entrance region of the hippocampal formation, meaning it works as a pre-processor of received information by encoding this information and preparing it for further processing in the Ammon's Horn (30). The dentate gyrus is essential to the behavioral discrimination of similar spatial components of memory functions. This means that cells of the dentate gyrus have different spatial firing patterns, based on different spatial stimuli (31). In this way, the dentate gyrus contributes significantly to VSM tasks. Thus, we would expect to find a relationship between a larger volume of these subregions and better performance in the memory tasks utilized in the present study.

When analyzing variables that could be associated with our dependent variable, we observed the moderating role of age, when age was correlated with different hippocampal subregions. A negative correlation between age and volumetric data was expected. Therefore, a negative correlation would also be expected between age and the VSM index, since a volumetric reduction in hippocampal subregions may affect an individual's ability to perform learning and memory activities. Several studies show atrophy of the hippocampal formation in patients with dementia and, especially, in Alzheimer's dementia (32,33). We found a negative correlation between age and the right parasubiculum volume. However, we did not find a negative correlation between age and VSM index. This means that the data in our study showed that age could have an important effect on volumetric data, but not on behavioral performance. A possible explanation for this includes the relatively young mean age of the participants: the average age of approximately 35 years is far from the advanced age at which the first signs of cognitive decline are typically reported (34), although some subjects had already shown a decrease in their right parasubiculum volume at young ages in our study.

We also clarified the role of IQ by correlating it with the variables of volume and memory performance. As expected, IQ was positively correlated with the VSM index. In addition, when IQ was inserted into multiple linear regression models, it was significant for both hemispheres. These results were in line with our initial hypothesis and reinforced the notion that IQ is an essential variable in explaining and predicting memory (35). This makes sense because IQ is an estimate of the global intellectual level, which includes memory functions (36).

Our study had several limitations that limit generalizability. First, this study did not include any cortical regions, although cortical structures may contribute to the association investigated in this study. The decision to focus on bilateral hippocampus analyses contributed to a more specific study, and allowed us to investigate hippocampal structures and their subdivisions in a deeper manner. Also, the fact that the subjects participating in our study did not have any previous training in memory skills could explain contrasting differences found in hippocampal volumes, compared with control subjects. This may demonstrate that significant differences in hippocampal volume require not only naturally better memory abilities among the subjects studied, but far superior abilities or a long period of intense and specific memory skills training.

In addition, it is important to highlight methodological differences between the British study series and the present study related to this previous training in memory skills and also related to the visuospatial memory assessment. The memory performance of taxi drivers was evaluated in the first study of the British series only according to the amount of time a driver had spent as a taxi driver (10) and, in the second study, using a virtual reality town through which subjects had to navigate (13). This manner of assessing memory skills has higher ecological validity, meaning it is more similar to the daily tasks of the subjects. This differs from the evaluation used in our study, with traditional and computerized memory tests, and therefore could contribute to differences in the results. Another limitation of this study is the small sample size and the large number of predictor variables in the models. However, it is common for neuroimaging studies to have a similarly small sample size given the high costs of the procedure, among other reasons.

Lastly, an important limitation was the length of time between neuropsychological assessment and MRI examination (on average, 170 days in this study), since the brain is constantly changing due to brain plasticity and new synapses (37). Regarding this, we evaluated how many participants had a time difference equal to or less than 20 days, and extra analyses were done only with these subjects (n=12). Even so, we did not find significant results when correlating the total VSM index of these subjects with the bilateral anterior (head) and posterior (tail) hippocampal volumes. In addition, we did not find a significant correlation with the total hippocampal volumes for each hemisphere, nor when these volumes were directly correlated with the raw scores from the variables that composed the index (according to the EFA).

Finally, we can conclude that the volume of the hippocampal formations, bilaterally, was involved in mnemonic processes related to visuospatial memory. Specifically, the granular cells of the molecular layer and the CA4 were the hippocampal subregions that most contributed to performance on visuospatial memory tasks. Also, VSM processing was essential for a diverse set of daily activities and may be influenced by demographic variables, especially IQ, in healthy subjects.

## Supplementary material

Click here to view [pdf].
